# Residents' perceptions of a night float system

**DOI:** 10.1186/1472-6920-9-52

**Published:** 2009-08-03

**Authors:** Harish Jasti, Barbara H Hanusa, Galen E Switzer, Rosanne Granieri, Michael Elnicki

**Affiliations:** 1 Division of General Internal Medicine, Department of Medicine, University of Pittsburgh, Pittsburgh, PA, USA; 2 VA Center for Health Equity Research and Promotion, VA Pittsburgh Healthcare System and Department of Psychiatry, University of Pittsburgh, Pittsburgh, PA, USA; 3 VA Center for Health Equity Research and Promotion, VA Pittsburgh Healthcare System and Departments of Psychiatry and Medicine and the Center for Research on Health Care, University of Pittsburgh, Pittsburgh, PA, USA

## Abstract

**Background:**

A Night Float (NF) system has been implemented by many institutions to address increasing concerns about residents' work hours. The purpose of our study was to examine the perceptions of residents towards a NF system.

**Methods:**

A 115-item questionnaire was developed to assess residents' perceptions of the NF rotation as compared with a regular call month. The categories included patient care, education, medical errors, and overall satisfaction. Internal Medicine housestaff (post-graduate years 1–3) from three hospital settings at the University of Pittsburgh completed the questionnaire.

**Results:**

The response rate was 90% (n = 149). Of these, 74 had completed the NF rotation. The housestaff felt that the quality of patient care was improved because of NF (41% agreed and 18% disagreed). A majority also felt that better care was provided by a rested physician in spite of being less familiar with the patient (46% agreed and 21% disagreed). Most felt that there was less emphasis on education (65%) and more emphasis on service (52%) during NF. Overall, the residents felt more rested during their call months (83%) and strongly supported the 80-hour workweek requirement (77%).

**Conclusion:**

Housestaff felt that the overall quality of patient care was improved by a NF system. The perceived improved quality of care by a rested physician coupled with a perceived decrease in the emphasis on education may have significant implications in housestaff training.

## Background

Resident work hour regulations are a major concern in residency training programs. Previous studies have noted the risks inherent in daytime sleepiness during the post-call period, including potential adverse patient outcomes [1,2]. Howard et al. demonstrated that residents' self-perceptions of their degree of physiologic sleepiness were poor and that levels approached those of clinical sleep disorders [3]. Additional studies have shown the negative impact of sleep deprivation and the effects on decision making and memory [4].

Due to these concerns, the American College of Graduate Medical Education (ACGME) has mandated limits for resident work hours [5]. A night float (NF) system has been implemented in many institutions to address these concerns and to help in achieving this goal [6-8]. The daytime physicians are relieved by a night team that admits patients and takes care of patient-related tasks. The day team then returns the following day to continue the care of the patients. Thus, the extended hours of the post-call day are avoided.

Although NF is a potential solution, it has generated a number of concerns. Residents feel that NF does not provide adequate teaching and view the rotation as more of a "service" rotation rather than as a learning opportunity [9-16]. Another concern is the discontinuity of care, which may result in poor patient satisfaction and adverse outcomes [17-23].

Many studies examining the perceptions of residents towards the NF system have been limited by small sample sizes ranging from 10 to 24 residents, brief surveys consisting of 10 to 30 questions, experiences of a group of residents in a single hospital or a single post-graduate year, and a lack of comparison between a NF and a non-NF system.

Our study proposes to address these limitations and further contribute to the continuing dialogue about the NF system. Our aim of the study was to assess the perceptions and opinions of residents towards the NF rotation as compared with a traditional call month rotation in the areas of patient care, medical errors, resident education, and overall satisfaction. We also examined differences by resident post-graduate years 1–4 since the resident's individual responsibilities might differ by seniority.

## Methods

A literature review was performed, using Medline to identify studies conducted between 1980–2004, to categorize overall domains and specific items relevant to NF issues. The general domains included patient care, the working environment, medical errors, resident education and learning environment, interpersonal skills and professionalism, and overall resident satisfaction. Individual interviews, using a uniform list of 15 open-ended questions as the basis for discussions and lasting 30–60 minutes, were then conducted with five residents and two fellows. New questions were generated and existing ones modified using the feedback from these interviews. Subsequently, a three-resident group discussion was conducted with three separate groups, which led to further modifications of the items.

The questionnaire was evaluated for face validity by six faculty members, two residents, and two fellows, all from our institution, and two residents and two fellows from outside institutions. The individuals from the outside institutions all had at least three years of experience with a NF system. On the basis of their comments, further modifications to the number of questions, the wording, and overall format of the questionnaire were made.

The final questionnaire consisted of 115 items and was divided into the six general domains [see Additional file 1]. Both positive and negative items were utilized and employed a Likert response scale in which 5 = strongly agree, 4 = agree, 3 = neither agree nor disagree, 2 = disagree, and 1 = strongly disagree. The survey also contained five open-ended questions to further explore each resident's specific opinions. Internal Medicine housestaff (post-graduate years 1–4) from three different teaching hospital settings (university, Veterans Administration, and community) at the University of Pittsburgh completed the questionnaire.

The survey was an anonymous questionnaire, and was approved and given an "exempt" status by the University of Pittsburgh Institutional Review Board, prior to study initiation.

### Data Analysis

Patterns of distribution, basic frequencies and mean scores were compared and analyzed by postgraduate year of training and completion of the NF rotation. The Mann-Whitney test was used to compare the mean scores of interns and residents. Correlations among individual items were examined using Spearman's rho. Analyses were performed using SPSS for Windows, version 11.5.

## Results

The response rate was 90% (n = 149), with 69 interns, 77 residents (PGY 2 and 3), and 3 chief residents (PGY 4). Of the respondents, 74 (50%) had completed the NF rotation (39 interns and 35 residents). Almost all of the responses, in the areas of patient care, medical errors, and resident education and satisfaction, had no significant differences between those who had and had not completed the NF rotation.

The mean ratings for selected statements in each area are shown in Tables 1, 2 &3. The individual means of interns and residents are shown when there was a significant difference between the two groups.

**Table 1:** Patient Care

**Item**	**Overall** **(SD)**
Better care provided by a rested physician less familiar with the patient	3.3 (.93)
Better care provided by a tired physician familiar with the patient	2.8 (.94)
Shared decision-making between the day team and the NF team improves patient care	3.2 (.89)
Continuity of patient care maintained	2.9 (.93)
Physician-patient relationship worsened	2.5 (.90)
Fewer adverse patient outcomes	3.1 (.72)
Overall quality of patient care improved	3.3 (.86)

No items with differences in means between interns and residents were significant at p < 0.05

**Table 2:** Education

**Item**	**Overall** **(SD)**
More emphasis on education	2.1 (.90)
More emphasis on service	3.5 (.97)
More opportunities for learning	2.8 (.92)
Less likely to learn about full impact of patient interventions	3.4 (1.0)
Less likely to learn about evolution of disease processes*	3.5 (.99)
Education impaired by:	
Fatigue	3.3 (1.0)
Lack of conferences	3.4 (1.0)
Absence of an attending physician	3.6 (.93)
Learning environment improved by:	
Scheduled night time conferences	2.5 (1.1)
Scheduled night time attending rounds	2.6 (1.2)
"Evening report" (to start before the NF shift)	3.0 (1.2)
Independent study with computer-based curriculum	3.0 (1.1)

* Means – Interns (3.2) and Residents (3.8); p = .002

Remaining items did not have significant differences in means between interns and residents

**Table 3:** Satisfaction

**Item**	**Overall** **(SD)**	**Intern**	**Resident**	**p**
Mood is better	3.5 (1.1)	3.9	3.2	<.001
Less stressed	3.6 (1.0)	3.9	3.4	.003
More well-rested	3.5 (1.1)	3.8	3.2	.001
More motivated	3.3 (1.1)	3.6	3.0	.003
Quality of one's time outside the hospital better	3.4 (1.2)	3.8	3.0	<.001
Family/personal life suffers	3.0 (1.1)	2.7	3.2	.016
More likely to develop a "shift-work" mentality	3.4 (1.0)	2.9	3.6	<.001
Feel less of a sense of responsibility	2.9 (1.1)	2.5	3.0	.009
More rested during call month because of NF	4.0 (.83)	4.3	3.9	.002
Learn more during call month because of NF	3.5 (.99)	3.7	3.4	-
Happier during call month because of NF	4.0 (.83)	4.2	3.7	<.001
Overall call month experience improved by NF	3.9 (.90)	4.2	3.6	<.001
Support 80-hour work week requirement	4.0 (1.1)	4.1	3.8	-
Prefer traditional overnight call system	2.5 (1.2)	2.1	2.9	<.001
Night float is a very valuable rotation	3.4 (1.0)	3.7	3.1	<.001

Significant p values shown for differences in means between interns and residents

### Patient Care

A majority felt that better care was provided by a rested physician in spite of being less familiar with the patient (46% agreed; 21% disagreed). Conversely, 25% thought that better care was provided by a tired physician who was more familiar with the patient, while 42% disagreed. More residents believed that the shared decision-making between the day and night teams improved patient care (39% agreed; 22% disagreed). About one-third of the respondents agreed (32%) that continuity of patient care was maintained during the NF rotation, while 33% disagreed. A significant number disagreed (57%) that the patient-physician relationship was worsened (17% agreed) and thought that there were fewer adverse outcomes (29% agreed; 16% disagreed). Overall, the housestaff felt that the quality of patient care was improved because of NF (41% agreed; 18% disagreed).

### Education

A majority of the respondents felt that, compared to a traditional call month, the NF rotation had less emphasis on education (65% agreed; 5% disagreed) and more emphasis on service (52% agreed; 11% disagreed) and therefore had fewer opportunities for learning (37% agreed; 21% disagreed). More felt that there were fewer opportunities to learn about the full impact of their interventions for the patients (49% agreed; 22% disagreed). Although most were concerned that they were less likely to learn about the evolution of disease processes (55% agreed; 18% disagreed), there was a significant difference in agreement between the residents and interns (35% and 20%, respectively; p = .002). This lack of continuity about the development of the medical conditions and the lack of feedback on their interventions were also emphasized in many of the responses to the open-ended questions. Education was thought to be impaired by fatigue (48% agreed; 26% disagreed), lack of conferences (54% agreed; 25% disagreed), and the absence of an attending physician (60% agreed; 13% disagreed). Interestingly however, fewer felt that the learning environment could be improved by night time attending rounds (29% agreed; 55% disagreed) or conferences (24% agreed; 57% disagreed). Rather, they seemed to prefer an "evening report" (to have before the NF shift) (45% agreed; 38% disagreed) or independent study with a computer-based curriculum (43% agreed; 34% disagreed).

### Medical Errors

Residents perceived that more medical errors were made, by them or someone else, during the traditional call night situation as compared with the NF setting, due to fatigue from lack of sleep (61% and 1%, respectively; 27% about equal) or fatigue from an excessive work load (59% and 1%, respectively; 35% about equal). Inadequate supervision by attendings was also considered to be a contributing factor to errors made during the NF setting (noted in the open-ended responses as well). Overall, most felt that an equivalent number of medical errors were made, either during a traditional call night or NF, with respect to ancillary or nursing support, availability of laboratory or radiological tests, and medical procedures.

### Overall Satisfaction

The opinions of interns and residents showed striking differences in the area of overall satisfaction. The differences in the means and proportions between interns and residents are shown in Tables 3 and 4.

**Table 4:** Satisfaction (% Intern/Resident Differences)

**Item**	**Intern** **(A/D) (%)**	**Resident** **(A/D) (%)**	**Total** **(A/D) (%)**	**p**
Mood is better	36/6	28/15	64/21	<.001
Less stressed	35/5	31/9	66/14	.003
More well-rested	35/7	30/15	65/22	.001
More motivated	25/7	18/16	43/23	.003
Quality of one's time outside the hospital better	32/8	24/20	56/28	<.001
Family/personal life suffers	12/23	23/17	35/40	.016
More likely to develop a "shift-work" mentality	18/18	36/7	54/25	<.001
Feel less of a sense of responsibility	9/27	23/22	32/49	.009
More rested during call month because of NF	43/2	40/4	83/6	.002
Learn more during call month because of NF	28/7	25/9	54/16	-
Happier during call month because of NF	41/0	35/6	76/6	<.001
Overall call month experience improved by NF	42/2	30/7	72/9	<.001
Support 80-hour work week requirement	40/4	37/10	77/14	-
Prefer traditional overnight call system	9/34	19/26	28/60	<.001
Night float is a very valuable rotation	31/4	19/12	50/16	<.001

A = agree; D = disagree

Compared to a call month, during their NF rotation, the residents were in a better mood (64% agreed; 21% disagreed), felt less stressed (66% agreed; 14% disagreed), and were more well-rested (65% agreed; 22% disagreed) and motivated (43% agreed; 23% disagreed). Although an almost equivalent number believed that family or personal life suffered during the NF rotation (35% agreed; 40% disagreed), a majority felt that the quality of their time outside the hospital was improved (56% agreed; 28% disagreed). Most also maintained a sense of responsibility for their patients (49% agreed; 32% disagreed).

A significant majority felt that, due to NF, they were more rested (83% agreed; 6% disagreed), "happier" (76% agreed; 6% disagreed), and learned more during their call month (54% agreed; 16% disagreed). Similarly, most felt that their overall traditional call month experience was improved because of NF (72% agreed; 9% disagreed).

A significant majority supported the 80-hour work week requirement (77% agreed; 14% disagreed); however, a substantial minority still preferred the traditional overnight call system (28% agreed; 60% disagreed). Overall, NF was felt to be a valuable rotation (50% agreed; 16% disagreed).

## Discussion

NF is a potential solution to fulfilling the work hour restrictions recently mandated by the ACGME. Our study examined residents' opinions in the fundamental areas of patient care, education, medical errors, and overall resident satisfaction.

The possible lack of continuity of patient care during the NF rotation is a highly debated concern. In our study, an almost equal number of residents either agreed or disagreed that continuity of care was compromised, further supporting the complexity of the issue. Several interventions may be implemented to improve continuity of care. Since shared decision-making was thought to improve care, enhancing the communication between the two teams will likely help maintain continuity. For example, standardized sign-out practices have been shown to reduce errors made by residents during the night-time [24]. Other strategies to improve continuity include pairing NF team members with members of the day team, all of whom would care for a specified patient population [25], and close oversight by attending physicians. These interventions will encourage professionalism and a personal investment in the care of the patient, areas argued to be deficient in the NF system.

The lack of emphasis on education during NF is another significant concern. As institutions initiate a NF system, specific educational prescriptions are vital components for the success of the program. As noted earlier, responses to the open-ended questions in our survey suggest that the residents are concerned about the lack of feedback on their medical reasoning process and the lack of follow-up about the evolution of disease processes. Greater supervision and feedback by night-time attendings and an "evening report" (held during the beginning of each NF period) could perhaps help maintain continuity of care, provide feedback on the decisions made overnight, and clarify the development of disease processes. These interventions will also fulfill the recommendations of the Residency Review Committee (RRC), which require that "education in the context of [patient care] activities must be provided to each resident [during night float]."

In addition, a standard NF curriculum, focusing on conditions and problems that are frequently encountered during the night, might help educate residents and provide the formal conferences that were thought to be an essential but missing element of the NF experience. As Drs. Ende and Davidoff recommended, "viewing housestaff programs as enterprises for hospital-based service is increasingly unacceptable... an important step in recasting [the structure] will be the development of curricula" [26].

The area of resident satisfaction revealed significant differences between the responses of interns (PGY 1) and residents (PGYs 2 and 3). There are several possible explanations for these differences. Overall, interns favored NF since it seemed to improve the quality of life in and outside of the hospital. This preference might be reinforced by the interns' schedules of having more ward months and generally being busier than residents. In contrast, the residents have fewer ward months and perhaps perceive that their limited learning opportunities are being compromised, as noted by the open-ended responses in the education domain.

There might also be a "cultural" effect. Although the interns had experienced a non-NF system for the first half of the academic year, the residents had experienced the system for one or two years. Perhaps this greater familiarity with a non-NF system influenced them to favor the traditional overnight call system. Lastly, the job functions of the interns and residents are different at our institution. Interns mainly cross-cover on patients already in the hospital, whereas residents admit new patients. This difference in the educational experience may also effect overall satisfaction.

### Limitations

Our study has several limitations that need to be acknowledged. Our questionnaire was designed to be the initial step towards the future development of a standardized instrument and thus did not include all the steps for ensuring reliability and validity. This process will be undertaken in future work. In addition, our survey did not collect socio-demographic information, which can potentially influence residents' perceptions. Finally, the respondents were from one institution with a specific type of NF rotation. The NF system at other institutions might be different, thereby limiting the generalizability of our results.

### Strengths

Compared to previous smaller studies, our detailed questionnaire (115 items) examining multiple areas and the high response rate (90%) provides a more accurate picture of the perceptions of housestaff towards the NF system. In addition, it incorporates the opinions of both interns and residents in different hospital environments, and those who had experienced both NF and non-NF systems. This is in contrast to other studies that had conducted surveys of residents who did not experience a non-NF system. Lastly, our study has been conducted at a time when the NF system and the associated working and educational environments have significantly changed since the time of many other studies. For example, there has been an increased incorporation of technology (e.g. computer-based order entry), increased ancillary support, and changes in residency educational programs, all factors which can significantly impact the residents' perceptions of the NF system.

## Conclusion

The night float system is a practical intervention towards meeting the resident work hour limitations. However, the lack of emphasis on education and reduction in continuity of patient care were significant concerns. Novel clinical and educational interventions are essential to address these deficiencies and thereby improve the quality of patient care, enrich resident education, and promote overall satisfaction.

## Competing interests

The authors declare that they have no competing interests.

## Authors' contributions

All authors participated in the design, coordination, and analysis of the study and helped to draft the manuscript. All authors have also read and approved the final manuscript.

## Pre-publication history

The pre-publication history for this paper can be accessed here:



## Supplementary Material

Additional file 1 **Night float evaluation survey**. This is the survey that was distributed to the housestaff. Click here for file
