# Supplementary material for: Residents' perceptions of a night float system
Source: BMC Med Educ. 2009 Aug 3;9:52. doi: 10.1186/1472-6920-9-52 (PMC2728710; doi:10.1186/1472-6920-9-52)
Supplement: Additional file 1 — Night float evaluation survey. This is the survey that was distributed to the housestaff. [file 1472-6920-9-52-S1.pdf]

The Night Float Rotation Evaluation Survey

Preliminary Information

Please indicate your level of training:

____ Intern (PGY-1)
____ Junior Resident (PGY-2)
____ Senior Resident (PGY-3)
____Other (specify) _____________

Have you done the night float rotation?

_____ **No**. Select a hospital whose night float rotation you are most familiar with to use as a comparison to your call month experience when filling out the survey.

____MUH ____VA ____Shadyside

_____ **Yes**. Please specify the location. If you have completed the night float rotation at more than one hospital, please select only **one** hospital to use as a comparison to your call month experience when filling out the survey.

____MUH ____VA ____Shadyside

I. Patient Care

Compared to <u>nights</u> on an average call month, during the night float rotation:	Strongly Disagree	Disagree	About Equal	Agree	Strongly Agree
The overall quality of patient care is improved	1	2	3	4	5
Better care is provided by a rested physician who is less familiar with the patient	1	2	3	4	5
Better care is provided by a tired physician who is familiar with the patient	1	2	3	4	5
The shared decision-making between the day team and the night float team improves patient care	1	2	3	4	5
Continuity of patient care is maintained	1	2	3	4	5
"Sign-out" about previously admitted patients is adequate	1	2	3	4	5
The physician-patient relationship is worsened	1	2	3	4	5
Patients are more confused as to who is their primary physician	1	2	3	4	5
Patients are more satisfied with their care	1	2	3	4	5
Overall patient care is less costly	1	2	3	4	5
The length of stay for patients admitted overnight is decreased	1	2	3	4	5
There are fewer adverse patient outcomes	1	2	3	4	5

II. Personal Aspects

Compared to an average call month, during the night float rotation:	Strongly Disagree	Disagree	About Equal	Agree	Strongly Agree
One's mood is better	1	2	3	4	5
One feels less stressed	1	2	3	4	5
One feels less isolated	1	2	3	4	5
One is more well-rested	1	2	3	4	5
One is more motivated	1	2	3	4	5
The quality of one's time outside the hospital is better	1	2	3	4	5
Family/personal life suffers	1	2	3	4	5
One is more likely to develop a "shift-work" mentality	1	2	3	4	5
One feels less of a sense of responsibility	1	2	3	4	5

III. Working Environment/Resources

Compared to <u>nights</u> on an average call month, during the night float rotation:	Strongly Disagree	Disagree	About Equal	Agree	Strongly Agree
One is more efficient	1	2	3	4	5
One spends more time on writing progress notes and orders	1	2	3	4	5
One spends less time on non-patient related tasks	1	2	3	4	5
One orders fewer lab tests	1	2	3	4	5
One orders fewer consults	1	2	3	4	5
One manages health care resources more cost-effectively	1	2	3	4	5
Nursing staff is more likely to be available	1	2	3	4	5
Nursing/housestaff relationships are better	1	2	3	4	5
One has a more difficult time contacting the covering attendings	1	2	3	4	5
The day team signs out inappropriate tasks to be completed	1	2	3	4	5
The overall workload is generally less	1	2	3	4	5
The number of patients admitted per night is too many	1	2	3	4	5
The patient to intern ratio is less manageable	1	2	3	4	5
One has more autonomy	1	2	3	4	5

There is sufficient "backup"	1	2	3	4	5
If you **Disagree** or **Strongly Disagree** with the above statement, then there is **not** enough "back-up" from:					
Residents	1	2	3	4	5
Fellows	1	2	3	4	5
Attendings	1	2	3	4	5
Sub-specialty services (e.g. surgery, ortho)	1	2	3	4	5
Other (please specify):					

IV. Miscellaneous

	Strongly Disagree	Disagree	About Equal	Agree	Strongly Agree
With reference to our current night float system (residents admit; interns cross-cover):					
Keep it as it is	1	2	3	4	5
Interns should also admit patients (in addition to cross-coverage)	1	2	3	4	5
Residents should also cross-cover (in addition to admitting)	1	2	3	4	5
It should continue to count as a call month	1	2	3	4	5

If you have **done** the night float rotation:
On average, about how many hours of sleep per **night** did you get in the hospital?
On average, about how many hours of sleep did you get during the **day**?

V. Medical Errors

Please select the setting in which more medical errors have been made (by you or someone else) due to:

	Night Float	Call Night	About Equal	Neither
Fatigue from lack of sleep	1	2	3	4
Fatigue from excessive work load	1	2	3	4
Poor communication (sign-out) from day team	1	2	3	4
Inadequate clerical/administrative support	1	2	3	4
Inadequate ancillary support (i.e. phlebotomy, transport)	1	2	3	4
Inadequate nursing support	1	2	3	4
Inadequate supervision by residents	1	2	3	4
Inadequate supervision by attendings	1	2	3	4
Insufficient clinical knowledge	1	2	3	4
Incorrect prescribing of medications	1	2	3	4
Delay in ordering a diagnostic test	1	2	3	4
Delay in correcting abnormal electrolyte values	1	2	3	4
Delay in performing procedures that needed to be done	1	2	3	4
Decreased availability of radiological tests	1	2	3	4
Decreased availability of lab tests	1	2	3	4

VI. Learning Environment

Compared to <u>nights</u> on an average call month, during the night float rotation:	Strongly Disagree	Disagree	About Equal	Agree	Strongly Agree
There is more emphasis on education	1	2	3	4	5
There is more emphasis on service	1	2	3	4	5
One has more time for reading	1	2	3	4	5
One has more free time ("down-time")	1	2	3	4	5
One has more opportunities for learning	1	2	3	4	5
The range of diagnoses of newly admitted patients is adequate	1	2	3	4	5
Newly admitted patients have more acute issues	1	2	3	4	5
One is less likely to learn about the full impact of patient interventions	1	2	3	4	5
One is less likely to learn about the evolution of disease processes	1	2	3	4	5
There are more "inappropriate" admissions	1	2	3	4	5
If you answered **Agree** or **Strongly Agree** to the item above, please list the diagnoses designated as inappropriate admissions:					

There is ***sufficient*** teaching	1	2	3	4	5
If you **Disagree** or **Strongly Disagree** with the above statement, then there is **not** enough teaching by:					
Resident to intern	1	2	3	4	5
Attending to intern	1	2	3	4	5
Attending to resident	1	2	3	4	5
Other (please specify):					

Education is impaired by:					
Fatigue	1	2	3	4	5
Excessive work load	1	2	3	4	5
Lack of time	1	2	3	4	5
Lack of conferences	1	2	3	4	5
Absence of an attending physician	1	2	3	4	5
Other (please specify):					

The learning environment could be improved by:					
Scheduled night time attending rounds	1	2	3	4	5
Scheduled night time conferences	1	2	3	4	5
An "evening report" (to start before the night float shift)	1	2	3	4	5
Independent study with computer-based cases/curriculum	1	2	3	4	5

VII. Education

In general, I like:	Strongly Disagree	Disagree	About Equal	Agree	Strongly Agree
Independent study	1	2	3	4	5
Noon conferences	1	2	3	4	5
Attending rounds	1	2	3	4	5
Grand rounds	1	2	3	4	5
Morning report	1	2	3	4	5
Reading a textbook	1	2	3	4	5
Looking up things on UpToDate	1	2	3	4	5
Other (please specify):					

VIII. Interpersonal-Communication Skills

Compared to <u>nights</u> on an average call month, during the night float rotation:	Strongly Disagree	Disagree	About Equal	Agree	Strongly Agree
One is less likely to thoroughly explain things (e.g. exams, treatment options) to patients	1	2	3	4	5
One is less likely to use open-ended questions when interviewing patients	1	2	3	4	5
One is less likely to give patients an opportunity to talk and ask questions	1	2	3	4	5
One communicates more effectively with patients and their families	1	2	3	4	5
One is less likely to be empathetic towards the patients	1	2	3	4	5
One is not as sensitive to the patient’s immediate physical needs	1	2	3	4	5
One is not as sensitive to the patient’s immediate emotional needs	1	2	3	4	5
One is less able to put patients at ease	1	2	3	4	5
One is more likely to rush or spend too little time with the patients	1	2	3	4	5
One is less thorough in the workup of newly admitted patients	1	2	3	4	5
It is difficult to sign off the patients admitted overnight	1	2	3	4	5
It is more difficult to take care of the terminally ill	1	2	3	4	5
It is more difficult to obtain an appropriate informed consent	1	2	3	4	5
One faces more ethical dilemmas	1	2	3	4	5
It is more difficult to manage a patient who has been deemed incompetent	1	2	3	4	5

IX. Overall Thoughts

	Strongly Disagree	Disagree	About Equal	Agree	Strongly Agree
Overall, my call month experience is improved by night float	1	2	3	4	5
I am more rested during my call month because of night float	1	2	3	4	5
I learn more during my call month because of night float	1	2	3	4	5
I am happier during my call month because of night float	1	2	3	4	5
The night float rotation has more educational value compared with the overnight call system	1	2	3	4	5
Overall, the night float rotation is a very valuable rotation	1	2	3	4	5
I am satisfied with the current night float rotation	1	2	3	4	5
I prefer the traditional overnight call system	1	2	3	4	5
I support the 80-hour work week requirement	1	2	3	4	5

Please write down any additional concerns/thoughts that you might have with the night float rotation. For example, what are the best features? What would you like to change?

Thank you **very much** for completing the questionnaire.
